# Western corn rootworm abundance, injury to corn, and resistance to Cry3Bb1 in the local landscape of previous problem fields

**DOI:** 10.1371/journal.pone.0237094

**Published:** 2020-07-31

**Authors:** Coy R. St. Clair, Graham P. Head, Aaron J. Gassmann

**Affiliations:** 1 Department of Entomology, Iowa State University, Ames, IA, United States of America; 2 Bayer Crop Science, Resistance Management, Chesterfield, MO, United States of America; University of Tennessee, UNITED STATES

## Abstract

Western corn rootworm, *Diabrotica virgifera virgifera* LeConte (Coleoptera: Chrysomelidae), is a major pest of corn in the United States. Transgenic corn expressing insecticidal proteins derived from the bacterium *Bacillus thuringiensis* (Bt) is an important tool used to manage rootworm populations. However, field-evolved resistance to Bt threatens this technology. In areas where resistance is present, resistant individuals may travel from one field to a neighboring field, spreading resistance alleles. An important question that remains to be answered is the extent to which greater-than-expected root injury (i.e., >1 node of injury) to Cry3Bb1 corn from western corn rootworm is associated with rootworm abundance, root injury, and levels of resistance in neighboring fields. To address this question, fields with a history of greater-than-expected injury to Cry3Bb1 corn (focal fields) and surrounding fields (< 2.2 km from focal fields) were examined to quantify rootworm abundance, root injury, and resistance to Cry3Bb1 corn. Additionally, use of Bt corn and soil insecticide use for the previous six years were quantified for each field. Resistance to Cry3Bb1 was present in all fields assayed, even though focal fields had grown more Cry3 corn and less non-Bt corn than surrounding fields. This finding implies that some movement of resistance alleles had occurred between focal fields and surrounding fields. Overall, our data suggest that resistance to Cry3Bb1 in the landscape has been influenced by both local rootworm movement and field-level management tactics.

## Introduction

Transgenic crops that produce insecticidal proteins derived from the bacterium *Bacillus thuringiensis* (Bt) have been widely used since their introduction in the 1990’s, accounting for 100 million hectares of global cropland in 2017 [1]. These crops have provided a number of benefits to both farmers and the environment, including higher yields, reduced adverse impacts on non-target species, and reduced need for conventional insecticides [2–4]. However, field-evolved resistance, where a decrease in pest susceptibility to a toxin is caused by exposure to the toxin in the field, threatens to curtail these benefits [5].

One tactic used to delay field-evolved resistance to Bt crops is the refuge strategy, which entails planting non-Bt plants alongside Bt plants. Under the refuge strategy, Bt-susceptible insects surviving on non-Bt plants randomly mate with the relatively rare Bt-resistant individuals, producing heterozygotes [6]. This strategy works best when the dose of the Bt toxin is high enough that resistance is functionally recessive, when resistance alleles have a low initial frequency, and when resistance imposes a fitness cost (i.e., resistant individuals experience lower fitness in the absence of Bt than susceptible individuals) [7–10]. In addition, plants that produce multiple Bt toxins targeting the same pest (i.e., pyramids) can enhance the refuge strategy. When pyramids are used with a refuge, insects resistant to one toxin are killed by a second toxin, which can be more effective at delaying resistance than using single traits sequentially [11].

The refuge strategy operates on the level of the individual field, but the composition of the broader landscape can influence pest abundances and distributions [12,13]. In areas where the landscape consists largely of agricultural crops, fields planted adjacent to one another may provide large contiguous tracts of potential habitat for pest species; thus, pest movement behavior can play an important role in population structure and spatial prevalence as insects move among fields [14]. In particular, the evolution of field-evolved resistance to Bt may be affected by landscape composition and insect movement patterns [15–17]. While many studies have focused on field-level evaluations of pest populations and Bt resistance, studies examining larger scales are needed to understand pest population structures in agricultural landscapes.

Western corn rootworm (*Diabrotica virgifera virgifera* LeConte) is a univoltine coleopteran which has developed field-evolved resistance to all current Bt traits available to farmers for management of this pest [18–20]. High levels of rootworm abundance can result in significant economic losses, with each node of root pruning resulting in approximately 15 to 17% loss in yield [21,22]. Field-evolved resistance to the earliest Bt trait (Cry3Bb1, registered in 2003) [23] was first detected in 2009 [24]. While Bt resistance in this pest has garnered much attention, the influence of movement within the landscape on resistance development is relatively unclear.

In general, most adult corn rootworm movement occurs within a short distance (approximately 2 km or less) of the natal field [25,26]. Females move short distances before mating [27], but are more likely to engage in long-distance flights after mating compared to males [26]. Females are also more likely to fly long distances in response to high larval density compared to low density [28]. As the season progresses, rootworm may disperse in the local landscape to fields with corn that is producing silk and pollen [29]. In Europe, as many as one-third of adults have been observed leaving the natal field for nearby fields, independent of larval density [30]. In areas of high corn cultivation, where attractive habitat is localized and abundant, adults are more likely to engage in local dispersal [14].

Dispersal is the primary mechanism responsible for the spread of field-evolved resistance in pest insects [31]. Field-evolved resistance to Cry3Bb1 in western corn rootworm appeared at roughly the same time in separate locations in the midwestern United States, suggesting independent evolution of resistance [18,24,32,33]. However, there is also indirect evidence that western corn rootworm transport resistance alleles via dispersal. In Nebraska, it was found that populations from fields where Cry3 traits had never been grown still displayed adaptation to Cry3Bb1 in plant-based bioassays, suggesting that the alleles for resistance were transported by resistant individuals from other populations in the landscape [34]. Thus, western corn rootworm movement in the landscape likely influences the spatial structure of populations and resistance to Bt in the landscape, an important factor in considering mitigation tactics [35,36].

It is largely unknown how rootworm populations are structured in landscapes characterized by corn production, such as northeastern Iowa. In particular, an important question is the degree to which resistance to Bt is localized to fields where resistance has facilitated injury to corn, or if resistance is more widespread in the landscape. The goal of our study was to better understand the spatial structure of western corn rootworm in the landscape surrounding fields that had previously experienced injury to Bt corn (previous problem fields). We examined root injury to corn by rootworm, adult rootworm abundance, resistance to Cry3Bb1, and management history in problem fields and fields in the local landscape. We hypothesized 1) fields closer to previous problem fields would have higher root injury, rootworm abundance, and levels of adaptation to Cry3Bb1 than fields further away; and 2) previous problem fields would have different management histories for western corn rootworm compared to fields in the surrounding landscape.

## Materials and methods

### Field selection

**Fig 1:**
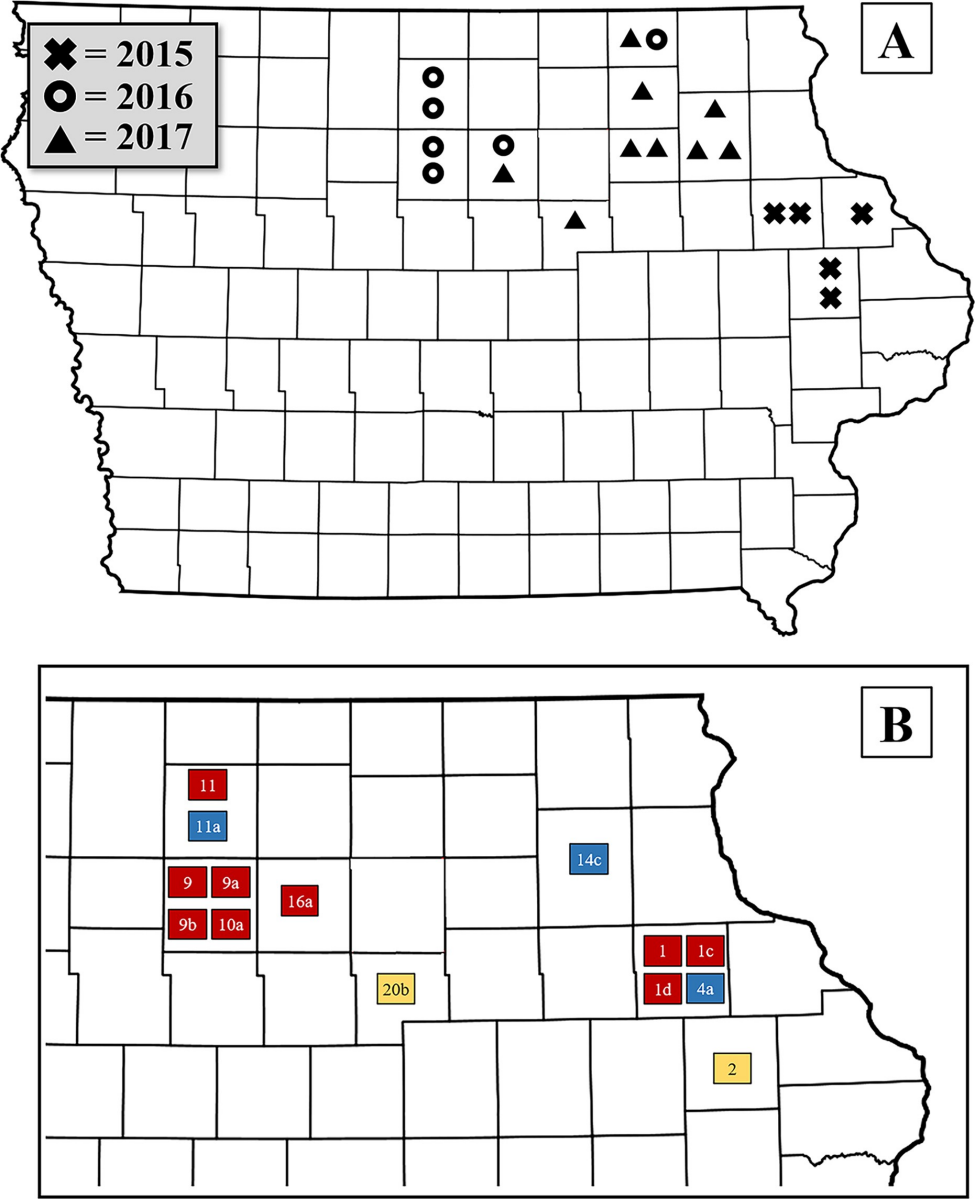
A) Map of Iowa, USA showing counties where focal fields were located and B) inset of northeastern Iowa showing populations assayed for resistance to Cry3Bb1. For A) Map of Iowa, each field had experienced greater-than-expected injury to Cry3Bb1 or mCry3A corn (> 1 node, node injury scale [NIS]) in at least one year between 2009 and 2013. Mean number of surrounding fields per focal field was 2.3. For B) inset of northeastern Iowa, fields with numerals only are focal fields, and fields with alphanumeric designation are surrounding fields that are associated with the focal field with the same numeral (e.g., field 9a is a field in the local landscape of focal field 9). Red squares indicate that the population experienced no difference in survival between non-Bt and Cry3Bb1 corn and no developmental delay. Yellow squares indicate that the population experienced either a difference in survival between the corn types, or a developmental delay on Cry3Bb1 corn, but not both. Blue squares indicate that the population showed a difference in survival and experienced a developmental delay on Cry3Bb1. See Figs 4 and 5 for details on bioassay results.

Over three years (2015, 2016, and 2017), a total of 20 fields with a history of greater than expected rootworm injury to Bt corn expressing a Cry3 toxin (i.e., greater than one node of injury to Cry3Bb1 or mCry3A corn) [37] were selected for study in north-central and north-eastern Iowa (referred to as “focal fields”). Sample sizes were N = 5, N = 6, and N = 9 for 2015, 2016, and 2017, respectively (Fig 1A). In addition to focal fields, neighboring fields in the local landscape were also selected for sampling (referred to as “surrounding fields”). We defined the “local” landscape as the area surrounding a focal field within approximately 2 km, and surrounding fields were selected based on this criterion. Sample sizes for surrounding fields were N = 12, N = 15, and N = 19 for 2015, 2016, and 2017, respectively. These fields were located a maximum of 2.2 km from the focal field (measured centroid to centroid), and the number of surrounding fields associated with a focal field ranged from 0 to 4 (mean = 2.3 surrounding fields/focal field). Verbal permission was obtained from land owners to sample at each field location. Because all research was conducted within the state of Iowa, no permits were required to collect insect pests from agricultural fields and bring them to a laboratory at Iowa State University for study.

### Rootworm abundance and root injury

Western corn rootworm abundance was measured using yellow sticky traps (Pherocon AM unbaited yellow sticky traps, Trécé Inc., Adair, OK) placed on the plant at ear-level. Ten traps were placed in each field in two parallel transects of five traps each following Dunbar et al. [38]. Traps were placed 30 m apart along transects, with ca. 15 m between transects, and both transects were set a minimum of 30 m from any field border. There were three consecutive sampling periods for each field, which ranged from six to 10 days (7.2 d ± 1.1 mean ± sd), and began 20 July in 2015 and 24 July in 2016 and 2017.

Root injury was assessed by digging roots of corn plants in the rows adjacent to where the sticky traps were placed, with 10 roots sampled per field. Roots were washed and rated on the 0–3 node injury scale within 48 hours of being sampled from the field [39]. Additionally, a sample of leaf tissue was collected from each plant at the time of digging and used to determine the Bt trait(s) present in the field using an ELISA-based test kit (Envirologix Inc., Portland, ME).

### Collection of adults and single-plant bioassays

All fields were sampled for live adult rootworm using a manual aspirator (BioQuip Products Inc., Rancho Dominguez, CA). The mean number of adults collected per field for all years was 45.8 ± 143.1 (mean ± sd). Of the 66 fields of the study, at least one adult rootworm was captured in 30 fields. Upon collection, adult rootworm were brought to the laboratory and placed in 18 × 18 × 18 cm plastic insect cages (MegaView Science Co. Ltd., Taichung, Taiwan), with rootworm from each field held in a separate cage. Cages were stored in an environmental chamber (I41-LL, Percival Scientific, Perry, IA) with 16:8h L:D cycle at 25°C, and ca. 65% RH. Each population was supplied with a corn leaf, Petri dish of complete adult diet (Frontier Agricultural Sciences, Newark, DE), and a 1.5% agar solid as a water source (Thermo Fisher Scientific Inc., Waltham, MA), all of which were replaced three times per week. Additionally, rootworm were provided with a Petri dish of oviposition substrate, consisting of finely sieved soil (particle size < 180 μm). Eggs were collected weekly until all adults were dead. Total number of eggs per field, from which rootworm were collected, was 1226.4 ± 3297.7 (mean ± sd).

Eggs were held in an environmental chamber for a minimum of two weeks, after which time they were stored at 4°C for at least five months to break diapause. Eggs were then removed from 4°C and placed in a dark environmental chamber at 25°C to induce hatching. Resulting larvae were used in a single-plant bioassay following Gassmann et al. [18]. The bioassay consisted of 12 replicates of two corn types, Bt (Cry3Bb1 [DKC 43–48] and its non-Bt genetic isoline [DKC 43–46]). Plants were grown in 1 L plastic cups (Placon, Madison, WI) to the V4-V5 growth stage [40]. Twelve neonate larvae (< 24h old) were placed gently on exposed root tissue using a paintbrush. The stalk of each plant was cut to approximately 20 cm and leaves were trimmed to approximately 10 cm to allow plants to fit within the environmental chamber. A thin layer of adhesive (Tanglefoot Insect Barrier, The Ortho Group, Marysville, OH) was applied to the inside rim of each bioassay cup to prevent larvae from escaping. Each plant was placed in an environmental chamber at 24°C, 16:8h L:D, 65% R.H, and received 50 mL of deionized water up to three times weekly if the soil surface was dry.

Seventeen days after larvae were added to plants, the remaining aboveground tissue was removed and the root mass, with potting medium and larvae, was placed on a Berlese funnel. The root mass remained on the funnel for four days, during which time the larvae were collected in a vial containing 85% ethanol. At the end of the fourth day, the vial was removed from the Berlese funnel and larvae were counted using a dissecting microscope (MZ6, Leica, Microsystems, Wetzlar, Germany). Head capsule width of larvae was measured using a digital microscope camera and imaging software (Moticam 2500, Motic Images Plus 3.0; Motic North America, Richmond, British Columbia, Canada). Larval head capsule widths were used to determine instar (first, second, or third) [41].

For 2015 field populations, bioassays began (i.e., larvae were placed on the first plant) 20 April, 2016, and ended (i.e., the last vial was collected from the Berlese funnel) on 14 June, 2016. For 2016 field populations, bioassays began 24 April and ended 9 July, 2017, and for 2017 populations, bioassays began 23 April and ended 10 June, 2018. Between one and three field populations were assayed weekly along with a Bt-susceptible, diapausing control strain. A control strain was assayed alongside each cohort (i.e., all field and control populations for which assays began in the same week) using identical procedures (i.e., 12 plants of each type per week, each of which received 12 larvae) to account for possible temporal variation in larval survival. There were five sets of controls run in all years, one for each cohort of bioassays. One set of controls in 2015 and one set in 2017 had low survival on non-Bt corn (mean < 15%), and were subsequently excluded, resulting in a total sample size of 48 Cry3Bb1 and 48 non-Bt plants in 2015 and 2017, and 60 of each type in 2016. Control populations were diapausing lab strains obtained from United States Department of Agriculture, Agricultural Research Service, North Central Agricultural Research Laboratory in Brookings, South Dakota. In 2015, controls used in bioassays consisted of four different populations: Control 1 (first collected in Finney Co., KS, 2000), Control 2 (Butler Co., NE, 1999), Control 3 (Moody Co., SD, 1986), and Control 4 (Phelps Co., NE, 1995). All control strains were collected and reared in a laboratory setting prior to 2003, which is the year when Bt corn was first introduced for management of western corn rootworm. Thus, the control populations used in this experiment had never experienced selection on Bt corn. Each strain was assayed once in 2015 alongside field populations as described above. In 2016 and 2017, controls assayed as part of each weekly cohort consisted of multiple replicates of a single population (Control 3 from 2015).

Fields were excluded from analysis if replication was below five plants of each corn type. In total, successful bioassays were conducted for two focal fields and three surrounding fields in 2015, two focal fields and four surrounding fields in 2016, and three surrounding fields in 2017 (14 total fields; Fig 1B). Sixteen fields from which adults were collected did not yield enough replicates to be included in the data analysis, because the number of adults (range = 0–140; mean ± sd = 11.9 ± 33.2) and eggs (range = 0–1500; mean ± sd = 296.3 ± 362.3) collected were too low. Fields for which replication was sufficient generally had a much higher number of adults (range = 35–897; mean ± sd = 202.5 ± 253.1) and eggs (range = 400–20,000; mean ± sd = 5442.9 ± 5337.0).

### Field management history

The total number of years of consecutive corn growth for each field was calculated using CropScape Data Layer (National Agricultural Statistics Service, United States Department of Agriculture; available at: https://nassgeodata.gmu.edu/CropScape). This metric was calculated from 2003 (inclusive), the year Bt corn was first marketed (e.g., for a field sampled in 2015, the maximum value for consecutive years of corn cultivation was 13). More detailed information on cropping history was also collected from crop consultants and farmers. Specifically, Bt traits used (Cry3Bb1, mCry3A, eCry3.1Ab, Cry34/35Ab1, or any pyramid thereof) and presence or absence of soil insecticide were determined for the year of sampling and the five previous years, for a total of six years of data. The number of years for which data could be obtained was 4.7 y ± 1.7 (mean ± sd) for Bt use, and 5.3 y ± 1.5 (mean ± sd) for soil insecticide use (S1–S3 Tables in S1 File). The following metrics were calculated from the information obtained from CropScape Data Layer and from crop consultants and farmers: 1) the total number of years the field was planted to corn continuously, 2) proportion of years the field was planted to corn in the previous six years (e.g., if a field was planted to corn in any three out of six years for which the management history was known, the proportion value would be 0.5), 3) proportion of years soil insecticide was used in years when corn was planted (e.g., if a field was planted to corn in four of the six years, and soil insecticide was used in two years when corn was planted, the proportion would be 0.5), 4) proportion of years non-Bt corn was planted, 5) proportion of years corn was planted that included a single-trait Cry3 protein (i.e., either Cry3Bb1 or mCry3A), 6) proportion of years single-trait Cry34/35Ab1 corn was planted, 7) proportion of years pyramided corn was planted (Cry34/35Ab1 + Cry3Bb1 or Cry34/35Ab1 + mCry3A), 8) proportion of years soil insecticide was used on Bt corn, and 9) proportion of years soil insecticide was used on non-Bt corn. No fields had planted corn expressing eCry3.1Ab.

### Data analysis

All data were analyzed using SAS 9.4 (SAS Institute Inc., Cary, NC, USA). For rootworm abundance, values used in analyses were calculated as [total number of adults caught/trap/sampling day] during the sampling period with the highest abundance for the field. To test the hypothesis that focal fields and surrounding fields differed in rootworm abundance and root injury, a mixed-model analysis of variance (PROC MIXED) was used. Either root injury or rootworm abundance was used as the response variable, with field type (focal field or surrounding field), year, and their interaction as fixed effects. Random effects in the model were field location (i.e., a focal field and its associated surrounding fields), location (year), field type (location), and field type (location (year)). Random terms were pooled in the model if P ≥ 0.25 for that term. Root injury and abundance were transformed using a square root transformation to improve normality of the residuals in analysis of variance. A Pearson’s correlation (PROC CORR) was used to examine the relationship between distance from focal field and either root injury or rootworm abundance (total N = 66 fields). Focal fields were included in the analysis, with a distance value of zero.

For root injury, in cases where a field was planted to Bt corn, refuge plants (i.e., those lacking a Bt trait in a field planted to a mixture of Bt corn and non-Bt corn) were removed from the analysis because refuge plants are expected to have greater levels of injury than Bt plants. Including refuge plants in analysis would inaccurately reflect injury to Bt corn in the field, thus these data were removed (mean number of refuge plants per field ± sd = 0.30 ± 0.60). However, a secondary analysis was conducted to assess injury to non-Bt corn compared to Bt corn for fields in which both types of corn were present (N = 17). A paired t-test (PROC TTEST) was performed to compare injury to Bt and non-Bt corn, with the null hypothesis that mean injury did not differ between Bt and non-Bt plants in these fields.

To analyze bioassay results, proportion survival on Cry3Bb1 corn and non-Bt corn was examined separately for each year using analysis of variance (PROC GLM). Proportion survival was the response, with population, corn type (Cry3Bb1 *vs* non-Bt), and their interaction as explanatory variables. Linear contrasts (CONTRAST statement) were used to compare proportion survival on Cry3Bb1 corn and non-Bt corn for each population to the susceptible controls within the year the assays were conducted. For Cry3Bb1 corn, significantly higher survival in the field population compared to the control populations would indicate resistance to Cry3Bb1. For survival on non-Bt corn, a difference between the field population and control populations would indicate some level of variation among populations for survival in the assay unrelated to the presence of Cry3Bb1 in corn. Proportion survival was transformed by the arcsine of the square root to improve normality of the residuals.

To further characterize resistance, survival on Cry3Bb1 corn was compared to survival on non-Bt corn within each population using a one-tailed t-test (PROC TTEST). A one-tailed test was used because it provided greater statistical power, as survival is expected to be lower on Cry3Bb1 corn than non-Bt corn or roughly equal, but not higher. Similarity in survival between the two corn types would indicate complete resistance to Cry3Bb1. Likewise, the proportion of third instar larvae (i.e., the number of larvae collected at the end of the assay that reached the third instar divided by the total number of larvae collected) was calculated for each corn type within a population, and a one-tailed t-test was used to test for a lower proportion of third instar larvae on Cry3Bb1 corn compared to non-Bt corn. Again, a one-tailed test was used because a smaller or equal proportion of third instar larvae would be expected on Cry3Bb1 corn compared to non-Bt corn, but not a higher proportion. A similar proportion of third instar larvae between the two corn types would indicate an equivalent developmental rate of the larvae, while a lower proportion of third instar larvae on Cry3Bb1 corn compared to non-Bt corn would indicate a slower developmental rate on Cry3Bb1 corn than non-Bt corn, and thus, incomplete resistance. Proportion survival and proportion of third instar larvae were transformed by the arcsine of the square root to improve normality of the residuals. T-tests used pooled variances in cases where variances were equal between the two groups being tested (e.g., survival in a population on Cry3Bb1 corn and survival in that population on non-Bt corn). In cases where variances were unequal, the Satterthwaite method was used. Control populations were not analyzed for instar, as very few larvae exposed to Cry3Bb1 survived the assay (mean proportion survival on CryBb1 corn in all controls = 0.05 ± 0.09 [mean ± sd]).

To account for variation in survival on non-Bt corn among the populations (see Results), a complementary analysis was conducted using corrected survival (proportion of surviving larvae on Cry3Bb1 corn in a replicate ÷ mean proportion surviving larvae on non-Bt corn in all replicates of a population) [42]. Analysis of variance (PROC GLM) was used to analyze corrected survival for each year separately. Corrected survival was the response variable and population was the independent variable. Linear contrasts were used (CONTRAST statement) to compare each field population to the controls. Significantly higher corrected survival in a field population compared to controls would indicate resistance to Cry3Bb1. To test for a difference in corrected survival between focal fields and surrounding fields, analysis of variance was used (PROC GLM) with corrected survival as the response variable and year, field type, and their interaction as independent variables.

To test whether field management differed for focal fields compared to surrounding fields in the year of sampling, a chi-square analysis was conducted (PROC FREQ). To test for a difference in historical management tactics, each field characteristic calculated as a proportion of management over the last six years was used in a mixed model analysis of variance (PROC MIXED), with field type (focal field or surrounding field) as a fixed effect, and field location (i.e., a focal field and its associated surrounding fields) and field type(location) as random effects. Random terms were pooled if P ≥ 0.25. Years of continuous corn was transformed by ln(y) to improve normality of the residuals. Sample sizes differed for each metric due to missing information for some fields (S1–S3 Tables in S1 File; see Table 2 for sample sizes).

For all field management metrics, multiple regression analysis (PROC REG) was used to test the effect of management strategies on rootworm abundance and root injury [43]. Independent variables for a field were excluded if the information being analyzed was unknown for more than three of the six years (S1–S3 Tables in S1 File). All metrics were first analyzed in a correlation matrix (PROC CORR) to determine if collinearity was present between any variables (Pearson’s correlation coefficient >0.80). Soil insecticide use in the year of sampling, proportion of years soil insecticide was used in the last 6 years, and the proportion of years soil insecticide was used on Bt corn in the last 6 years were collinear (S4 Table in S1 File). Collinearity indicates that the variation in the response could be potentially explained by other variables in the model, thus soil insecticide use in the year of sampling and proportion of years soil insecticide was used on Bt corn were removed from the analysis. The remaining variable represented the variation explained by all three collinear variables, and was a measure of overall soil insecticide use in the fields (referred to below as “soil insecticide use”). The following variables were then used as independent variables in the multiple regression, with either rootworm abundance or root injury as the response: non-Bt corn planted in the year of sampling (0 = no, 1 = yes), Cry3 corn planted in the year of sampling (0 = no, 1 = yes), Cry34/35Ab1 corn planted in the year of sampling (0 = no, 1 = yes), pyramided corn grown in the year of sampling (either Cry34/35Ab1 + Cry3Bb1 or Cry34/35Ab1 + mCry3A, 0 = no, 1 = yes), the total number of years the field was planted to corn continuously, the proportion of years the field was planted to corn in the previous six years, soil insecticide use, proportion of years non-Bt corn was planted, proportion of years single-trait Cry3 corn was planted (either Cry3Bb1 or mCry3a), proportion of years single-trait Cry34/35Ab1 corn was planted, and the proportion of years soil insecticide was used on non-Bt corn. In total, 47 fields were used in this analysis (S1–S3 Tables in S1 File). Stepwise selection was used to identify the most appropriate model (P<0.25 for inclusion in the model, P>0.15 for retention) following Dunbar et al. [38]. Rootworm abundance and root injury were transformed using a square root transformation to improve normality of the residuals.

## Results

### Rootworm abundance and root injury

**Fig 2:**
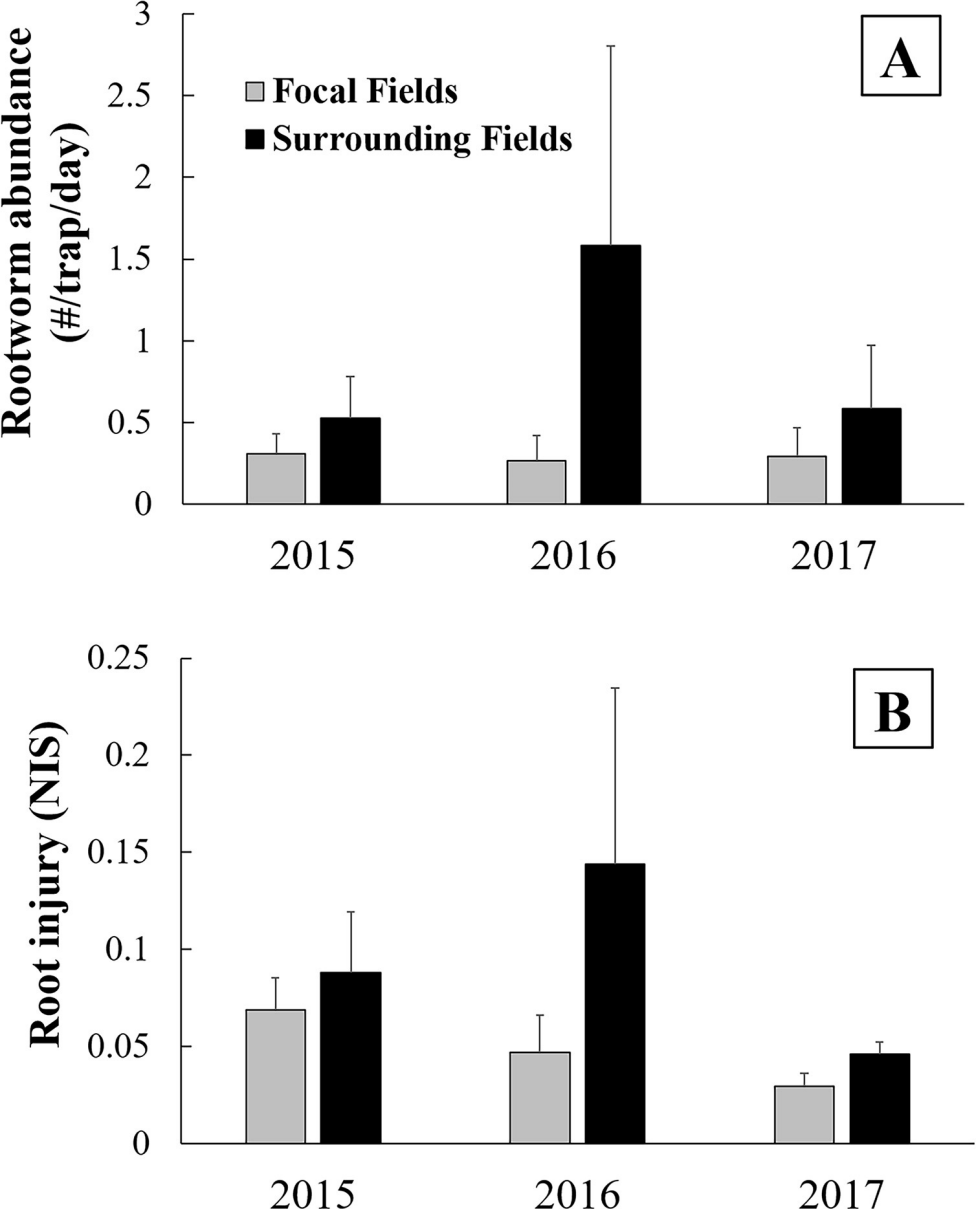
Abundance of western corn rootworm and root injury. A) Mean rootworm abundance for focal fields and surrounding fields, and B) mean root injury for focal fields and surrounding fields. Bar heights represent sample means and error bars are standard error of the mean.

**Fig 3:**
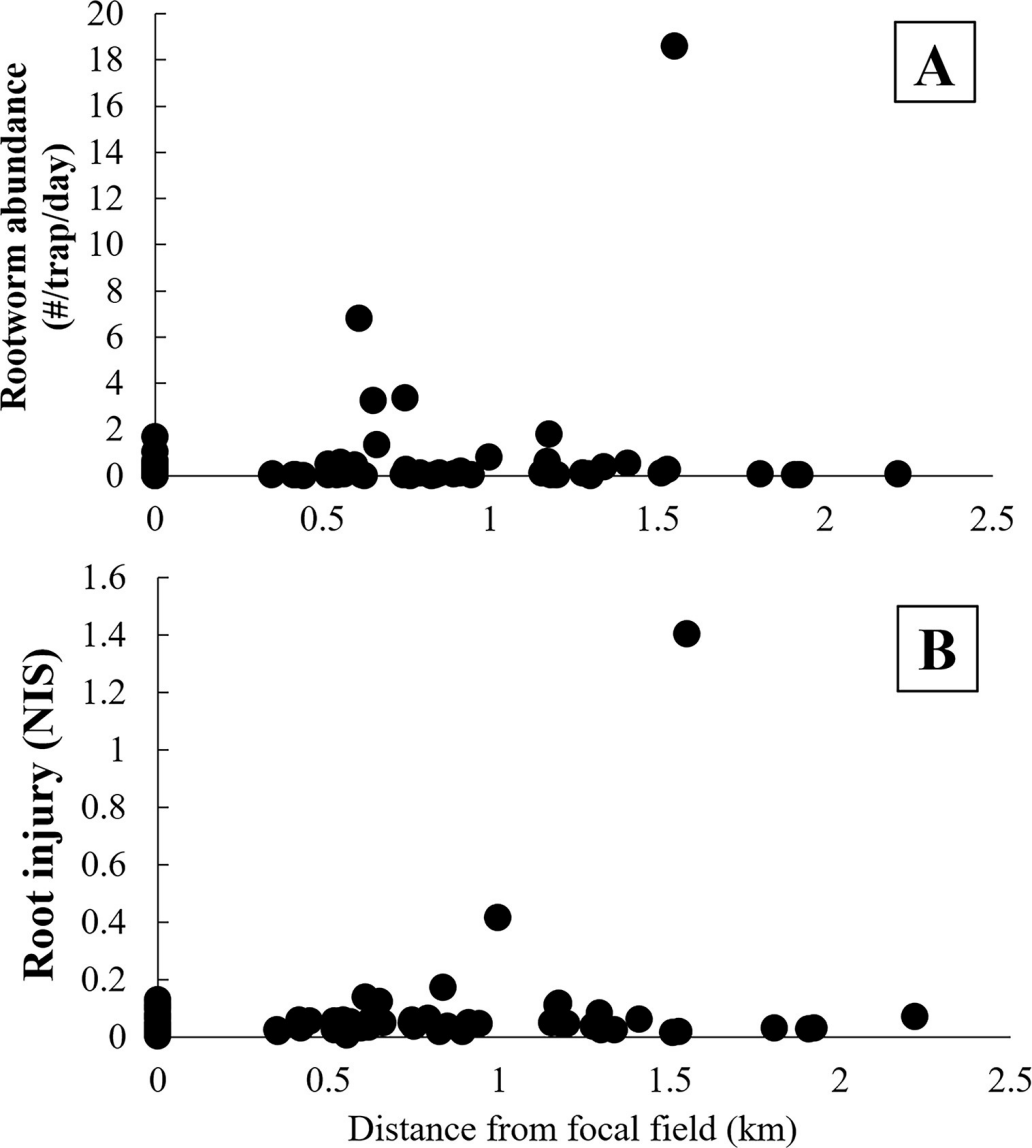
Scatterplot of western corn rootworm abundance and root injury with distance from focal field. A) Abundance of western corn rootworm and distance from focal fields and B) root injury and distance from focal fields. Rootworm abundance was not significantly correlated with distance from focal field in a Pearson’s correlation (r = 0.16, DF = 66, P = 0.20). Root injury was not significantly correlated with distance in a Pearson’s correlation (r = 0.2, DF = 66, P = 0.10).

Mean adult abundance for all focal fields was 0.29 rootworm/trap/day ± 0.09 (mean ± SE), and for all surrounding fields was 0.89 rootworm/trap/day ± 0.43 (mean ± SE). None of the factors tested (field type [focal field *vs* surrounding field], year, and their interaction) had a significant effect on rootworm abundance (Fig 2A; field type, F_1, 43_ = 0.55, P = 0.46; year, F_2, 43_ = 0.23, P = 0.79; field type × year, F_2, 43_ = 0.26, P = 0.77). Mean root injury in focal fields was 0.04 nodes ± 0.008 (mean ± SE), and for surrounding fields mean injury was 0.09 nodes ± 0.03. Likewise, none of the factors tested had a significant effect on root injury (Fig 2B; field type, F_1, 60_ = 1.39, P = 0.24; year, F_2, 60_ = 1.34, P = 0.27; field type × year, F_2, 60_ = 0.26, P = 0.77). For the 17 fields where refuge plants were found, there was not a statistical difference in injury to Bt corn compared to non-Bt corn (Bt mean ± SE = 0.04 ± 0.007, non-Bt mean ± SE = 0.05 ± 0.002; T = 0.56, DF = 16, P = 0.59). There was not a statistically significant correlation between distance from focal field and rootworm abundance (Fig 3A) or root injury (Fig 3B).

### Single-plant bioassays

**Fig 4:**
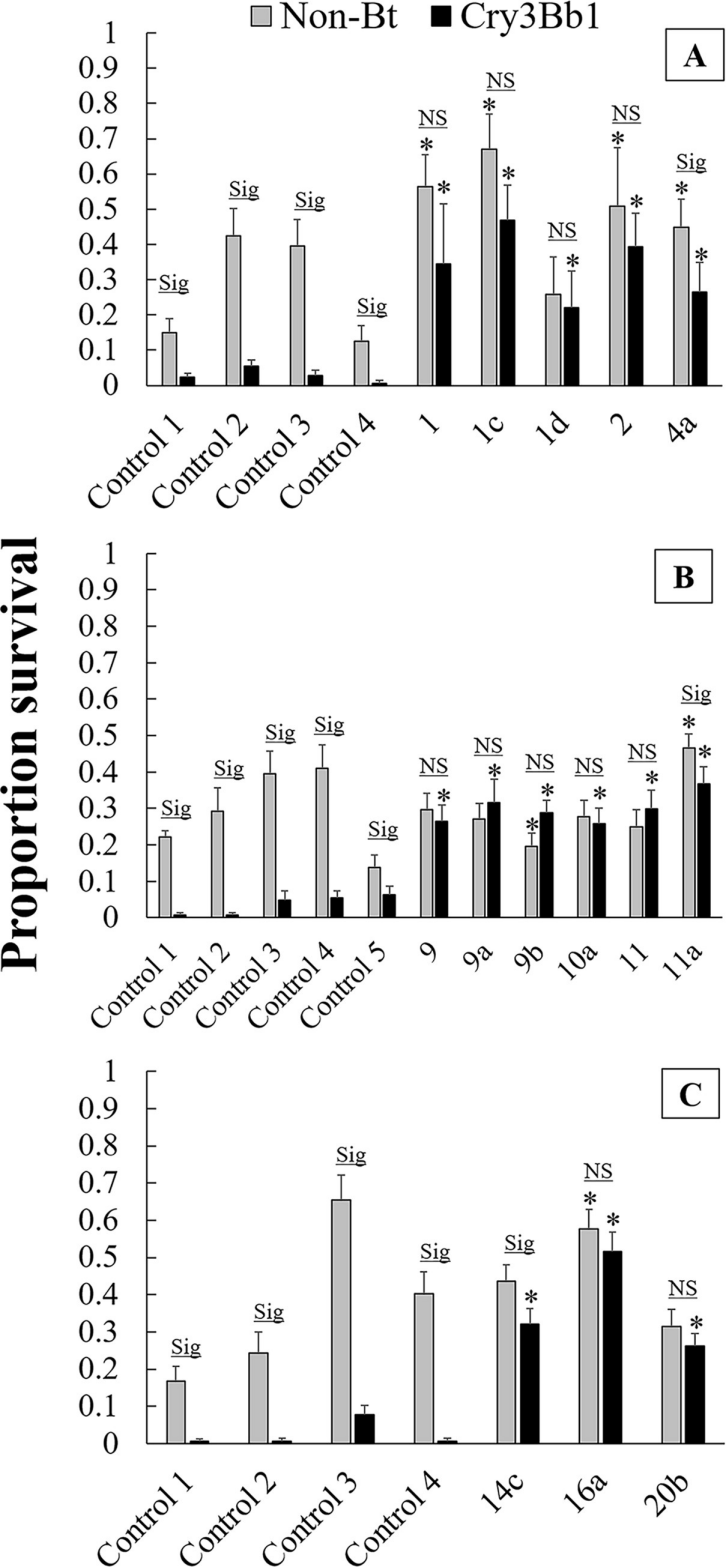
Proportion survival in single-plant bioassays on Cry3Bb1 corn and its non-Bt genetic isoline. A) 2015, B) 2016, and C) 2017. Bar heights represent sample means and error bars are standard error of the mean. For 2015, control populations were four diapausing lab strains that had never been exposed to Bt. For 2016 and 2017, control populations were replicates of Control 3 in 2015. Populations with numerals only originated from focal fields, and fields with alphanumeric designation originated from surrounding fields that are associated with the focal field with the same numeral (e.g., population 9a originated from a field in the local landscape of focal field 9). Bars with an asterisk were significantly different from controls on the same corn type (e.g., an asterisk above a black bar indicates a difference between survival on Cry3Bb1 corn in the field population compared to the controls). Sig. indicates a significant difference in survival between the two corn types within the same population, and NS indicates that no significant difference was found between survival on the two corn types.

For the bioassays, the interaction of population and corn type was significant in 2016 and 2017, indicating that survival differed between Cry3Bb1 and non-Bt corn, and this difference varied by population. The effect of population and corn type were both highly significant in 2015, while the interaction was not significant. All focal field and surrounding field populations tested had significantly higher survival on Cry3Bb1 compared to all controls of the same year in linear contrasts (Fig 4). Some variation existed with regard to survival on non-Bt corn; two focal fields and two surrounding fields in 2015 (fields 1, 1c, 2, and 4a), one surrounding field in 2016 (11a), and one surrounding field in 2017 (16a) had significantly higher survival on non-Bt compared to controls, and one surrounding field from 2016 (9b) had significantly lower survival on non-Bt compared to controls (Fig 4). A sign test showed that the number of field populations that had higher survival versus lower survival on non-Bt corn, compared to experimental controls, did not deviate from a null hypothesis that there were an equal number of populations with higher versus lower survival compared to controls (P = 0.13) [43].

All control populations showed significantly lower survival on Cry3Bb1 corn compared to non-Bt (Fig 4). Of the field populations tested (four focal fields and 10 surrounding fields) all focal fields and seven surrounding fields had equivalent survival on the two corn types. Only three populations had significantly lower survival on Cry3Bb1 corn compared to non-Bt corn: surrounding field 4a from 2015, surrounding field 11a from 2016, and surrounding field 14c from 2017 (Fig 4).

**Fig 5:**
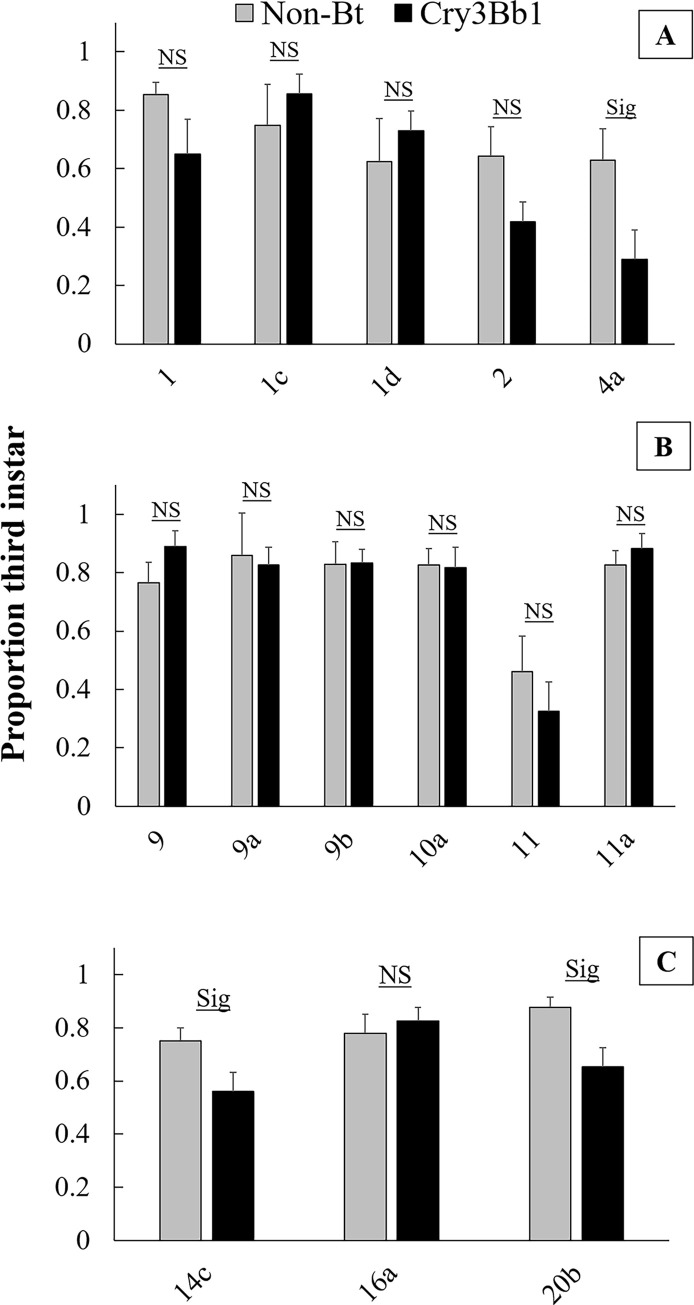
Proportion of third instar larvae in single-plant bioassays on Cry3Bb1 corn and its non-Bt genetic isoline. A) 2015, B) 2016, and C) 2017. Bars heights represent sample means and error bars are standard error of the mean. Sig. indicates a significant difference in the proportion of third instar larvae between the two corn types within the same population, and NS indicates that no significant difference in the number of third instar larvae was found between the two corn types.

There was no difference in proportion of third instar larvae between Cry3Bb1 corn and non-Bt corn for any of the focal field populations and seven of the ten surrounding field populations (Fig 5). Surrounding field 4a from 2015, surrounding field 14c from 2017, and surrounding field 20b from 2017 had a significantly lower proportion of third instar larvae on Cry3Bb1 corn compared to non-Bt corn.

Considering both differences in survival and development, most populations (all focal fields and six surrounding fields) demonstrated complete resistance to Cry3Bb1 corn (i.e., no difference in survival and no developmental delay). One population had a lower proportion of third instar larvae on Cry3Bb1, but did not show a difference in survival on the two corn types (20b). One population had lower survival on Cry3Bb1 corn compared to non-Bt, but did not show a developmental delay (11a). Two populations had lower survival on Cry3Bb1 and showed a lower proportion of third instar larvae, suggesting incomplete resistance to Cry3Bb1 (4a and 14c). These results reveal that, while all tested populations were resistant compared to susceptible controls, there was some variation in the level of adaptation to Cry3Bb1 in the landscape of northeastern Iowa (Fig 1B).

**Fig 6:**
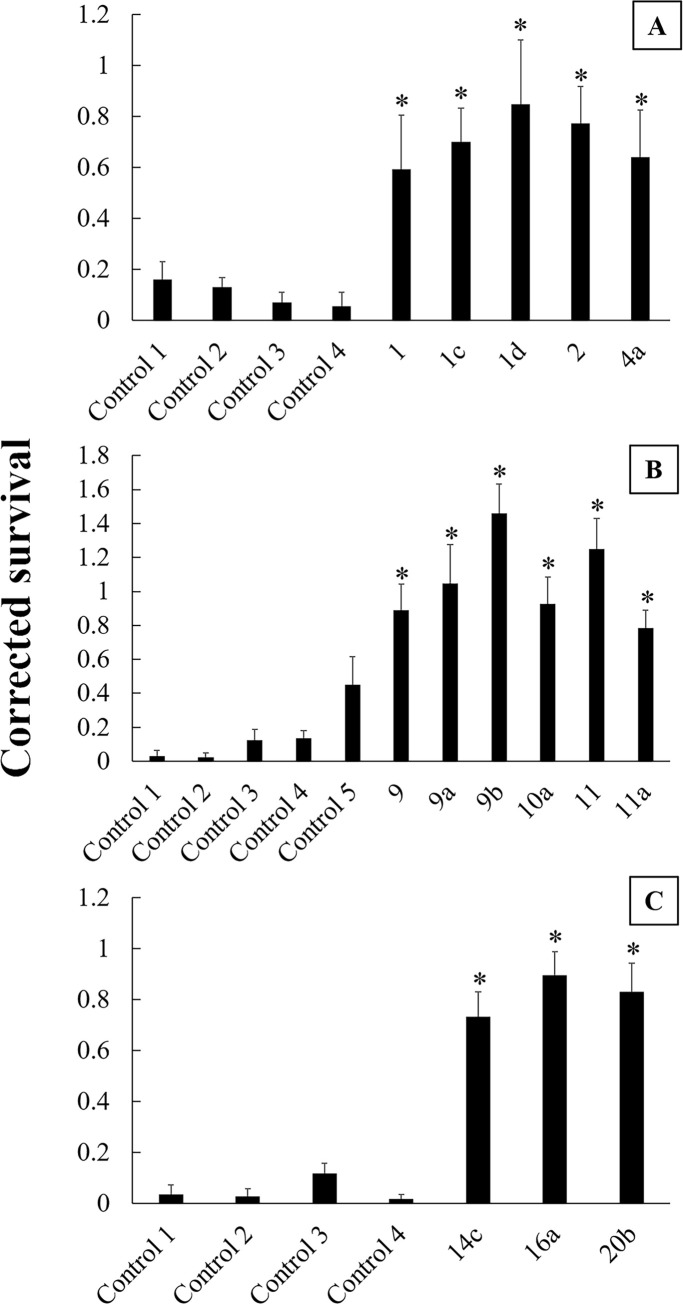
Corrected survival in single-plant bioassays. Bar heights are means and error bars are standard error of the mean. A) 2015, B) 2016, and C) 2017. Asterisks indicate that corrected survival in the population was significantly higher than the controls in the year the assay was conducted. For 2015, control populations were four diapausing lab strains that had never been exposed to Bt. For 2016 and 2017, control populations were replicates of Control 3 in 2015. Populations with numerals only originated from focal fields, and populations with alphanumeric designation originated from surrounding fields that are associated with the focal field with the same numeral (e.g., population 9a was collected from a field in the local landscape of focal field 9).

There was an effect of population on corrected survival in all years (Table 1). The linear contrasts used to compare corrected survival in each field population to the relevant control populations showed that all focal fields and all surrounding fields had significantly higher corrected survival compared to controls (Fig 6). Corrected survival did not differ between focal fields and surrounding fields (Table 2).

**Table 1:** Analysis of variance for survival and corrected survival in plant-based bioassays in each year.

		*Survival*			*Corrected Survival*		
*Year*	*Effect*	*DF*	*F*	*P*	*DF*	*F*	*P*
2015	Population ^a^	8, 169	14.98	<0.0001	8, 84	6.03	<0.0001
	Corn type ^d^	1, 169	44.42	<0.0001	-	-	-
	Pop. × Corn type	8, 169	1.84	0.07	-	-	-
2016	Population ^b^	10, 314	8.38	<0.0001	10, 167	9.96	<0.0001
	Corn type	1, 314	51.74	<0.0001	-	-	-
	Pop. × Corn type	10, 314	7.75	<0.0001	-	-	-
2017	Population ^c^	6, 203	26.55	<0.0001	6, 102	20.55	<0.0001
	Corn type	1, 203	115.17	<0.0001	-	-	-
	Pop. × Corn type	6, 203	10.8	<0.0001	-	-	-

^a^ 2015 populations: 4 control, 2 focal fields, 3 surrounding fields.

^b^ 2016 populations: 5 control replicates, 2 focal fields, 4 surrounding fields.

^c^ 2017 populations: 4 control replicates, 3 surrounding fields.

^d^ Corn type = Cry3Bb1 corn *vs* non-Bt corn.

**Table 2:** Analysis of variance for corrected survival with field type (focal fields *vs* surrounding fields), year of sampling, and their interaction as factors.

*Effect*	*DF*	*F*	*P*
Field Type ^a^	1, 204	0.07	0.79
Year ^b^	2, 204	4.29	**0.01**
Field Type × Year	1, 204	0.01	0.93

^**a**^ Field type = focal fields *vs* surrounding field.

^b^ Year = 2015, 2016, 2017.

### Field characteristics

Presence and type of Bt used and soil insecticide use did not differ between focal fields and surrounding fields for the year of sampling (Table 3). For historical management tactics, proportion of years non-Bt corn was planted was higher in surrounding fields compared to focal fields, and focal fields had planted more Cry3 corn than surrounding fields (Table 4).

**Table 3:** Chi-square analysis of field management tactics from the year a field was sampled.

*Field History Metric* ^*a*^	*Focal Fields* ^*b*^	*Surr*. *Fields* ^*b*^	*DF*	χ^2^	*P*
Non-Bt corn planted	0.15	0.26	1	0.98	0.32
Cry3 corn planted	0.25	0.09	1	3.15	0.08
Cry34/35Ab1 corn planted	0.00	0.07	1	1.37	0.24
Cry34/35Ab1 + Cry3 corn planted	0.60	0.59	1	0.01	0.92
Soil insecticide	0.25	0.30	1	0.20	0.65

^a^ Metrics are present or absent for the year the field was sampled. Absent = 0, Present = 1. For field categories, focal field = 0, surrounding field = 1.

^b^ Proportion of fields in each category using the management tactic.

**Table 4:** Mixed model analysis of variance and means of field history metrics of focal fields and surrounding fields from the past six years of management.

*Field History Metric* ^*a*^	*Focal Fields* ^*b*^	*Surr*. *Fields* ^*b*^	*DF*	*F*	*P*
Years cont. corn ^c^	5.05 ± 0.99 (20)	4.87 ± 0.65 (46)	1, 45	0.30	0.59
Proportion corn ^d^	0.83 ± 0.03 (20)	0.78 ± 0.03 (46)	1, 36	1.79	0.19
Proportion S.I. use ^e^	0.26 ± 0.07 (18)	0.22 ± 0.06 (37)	1, 35	0.83	0.37
Proportion non-Bt ^f^	0.09 ± 0.03 (15)	0.25 ± 0.05 (34)	1, 31	4.50	**0.04**
Proportion Cry3Bb1 or mCry3A ^g^	0.29 ± 0.07 (14)	0.11 ± 0.04 (33)	1, 30	9.58	**0.004**
Proportion Cry34/35Ab1 ^h^	0.01 ± 0.01 (14)	0.02 ± 0.01 (33)	1, 30	0.88	0.36
Proportion Cry34/35Ab1 + Cry3 ^i^	0.61 ± 0.08 (14)	0.62 ± 0.07 (33)	1, 30	0.02	0.90
Proportion S.I. on Bt ^j^	0.24 ± 0.08 (16)	0.20 ± 0.06 (34)	1, 32	1.05	0.31
Proportion S.I. on non-Bt ^k^	0.01 ± 0.01 (17)	0.03 ± 0.02 (37)	1, 35	0.58	0.45

^a^ Field history metrics are proportions of years taken from the most recent 6 years. S.I. indicates soil insecticide.

^b^ Mean ± SE (N).

^c^ Random factor: Location χ^2^ = 2.3, DF = 1, P = 0.06.

^d^ Random factor: Location χ^2^ = 0.0, DF = 1, P = 0.00; Location × Field type χ^2^ = 1.9, DF = 1, P = 0.08.

^e^ Random factor: Location χ^2^ = 7.4, DF = 1, P = 0.003.

^f^ Random factor: Location χ^2^ = 3.1, DF = 1, P = 0.04.

^g^ Random factor: Location χ^2^ = 6.7, DF = 1, P = 0.005.

^h^ Random factor: Location χ^2^ = 9.5, DF = 1, P = 0.002.

^i^ Random factor: Location χ^2^ = 6.5, DF = 1, P = 0.005.

^j^ Random factor: Location χ^2^ = 7.0, DF = 1, P = 0.004.

^k^ Random factor: Location χ^2^ = 1.3, DF = 1, P = 0.13.

Multiple regression revealed rootworm abundance to be negatively correlated with non-Bt corn planted in the year of sampling and soil insecticide use. There was a positive correlation between abundance and the historical proportion of Cry3 use, and this was the only metric that was significant at the P ≤ 0.05 level. These three independent variables explained approximately 26% of the observed variation in abundance (Table 5). Root injury was negatively correlated with soil insecticide use, and positively correlated with historical proportion of Cry3 use. The proportion of Cry3 was significant at the P ≤ 0.05 level, and these two variables explained approximately 24% of the observed variation in root injury (Table 5).

**Table 5:** Multiple regression for rootworm abundance and root injury using field management metrics and field type as possible parameters.

*Dependent Variable*	*Parameters*	*Slope*	*SE*	*F*	*P*	*Model r*^*2*^
Rootworm abundance (square root)	Non-Bt corn (year of sampling) ^a^	-0.46	0.25	3.35	0.07	0.27
	Proportion S.I. use ^b^ ^d^	-0.50	0.30	2.75	0.10	
	Proportion Cry3 ^c^	1.54	0.44	12.30	0.0011	
	(Intercept)	0.57	0.14	16.92	0.0002	
Root injury (square root)	Proportion S.I. use ^b^ ^d^	-0.10	0.07	2.04	0.16	0.24
	Proportion Cry3 ^c^	0.37	0.10	13.65	0.0006	
	(Intercept)	0.21	0.03	47.96	<0.0001	

^a^ Use of non-Bt corn in the year the field was sampled (0 = no, 1 = yes).

^b^ Proportion of years soil insecticide was used out of the most recent six years.

^c^ Proportion of years Cry3Bb1 or mCry3A were grown out of the most recent six years.

^d^ Variable was collinear with proportion of years soil insecticide was used on Bt corn and soil insecticide use in the years of sampling, which were removed as potential variables.

## Discussion

In our experiment, we found that rootworm abundance and root injury did not differ between fields with a history of injury to Cry3 corn and fields in the surrounding landscape (Fig 2). Additionally, we found that all field populations exhibited field-evolved resistance to Cry3Bb1 corn, and levels of resistance did not differ between focal fields and surrounding fields (Table 2, Figs 4–6). These findings indicate that we should reject our first hypothesis, that focal fields had higher rootworm abundance, root injury, and resistance to Cry3Bb1 than surrounding fields. However, our second hypothesis, that strategies for managing rootworm differed between focal fields and surrounding fields, was supported. We found that focal fields had grown more Cry3 corn and less non-Bt corn in the past six years compared to surrounding fields. Other studies have shown that selection pressure resulting from continuous planting of Cry3Bb1 corn in fields was associated with increased levels of resistance to this trait by western corn rootworm [24]. Because management differed in the two field types, but resistance to Cry3Bb1 did not, our data suggest that, to some extent, rootworm have carried alleles for resistance to Cry3Bb1 from focal fields to surrounding fields. However, evolution of Cry3 resistance in surrounding fields also likely occurred because Cry3 corn was planted in these fields.

The propensity for western corn rootworm to develop field-evolved resistance to Cry3Bb1 independently as a result of field-level selection is supported by multiple lines of evidence [24,31]. The refuge strategy is used by farmers to delay resistance to Bt, and has been utilized successfully to manage development of resistance in some pest species. In general, the success of the refuge strategy has hinged on a few important factors: low initial resistance allele frequency, the presence of fitness costs, and the meeting of the “high dose” requirement which renders resistance functionally recessive. When all or most of these factors are present, resistance may be delayed for extended periods of time [5]. Examples include European corn borer, *Ostrinia nubilalis*, pink bollworm, *Pectinophora gossypiella*, and tobacco budworm, *Heliothis virescens* [44–46]. Western corn rootworm, however, experience minor fitness costs of Bt resistance, are managed with Bt traits that do not meet the threshold for high-dose, and appear to have high initial frequency for Bt-resistance traits [32,35,47,48]. Thus, this pest is well-suited for adaptation to Bt and field-level selection may lead to rapid resistance evolution. There is, however, also evidence that Bt resistance is spread due to pest movement. For example, computer modeling work on *Helicoverpa zea*, has shown that clusters of up to 20 contiguous fields where Bt use was high led to rapid evolution of resistance due to pest movement [49]. Additionally, Cry3Bb1-resistant western corn rootworm populations in Nebraska were found in cornfields with no history of Cry3Bb1 selection [34]. Like many biological phenomena, there is probably no single explanation that could account for the observed distribution of resistance to Cry3Bb1 in the landscape; instead, it is likely influenced by a combination of independent evolution and allele movement among fields.

In our experiment, focal fields had grown more Cry3 corn in the past six years compared to surrounding fields, but levels of resistance in the two field types did not differ (Tables 2 and 4). One plausible explanation for this observation is that resistant rootworm moved from focal fields, where selection for resistance to Cry3Bb1 had been the most intense, into the surrounding landscape. This does not preclude that populations outside of focal fields were undergoing their own bouts of selection, but movement from focal fields would serve to bolster and homogenize resistance in the surrounding landscape [36,49]. This would explain why, in our experiment, previous management among focal fields and surrounding fields differed but rootworm resistance to Cry3Bb1 did not (Table 2, Fig 6). However, it is worth noting that an experiment in Nebraska, which also examined resistance to Cry3 in local landscapes, concluded that individual field-level selection was a major contributor to resistance levels [34]. Indeed, most fields in our experiment, focal fields and surrounding fields, had experienced some level of selection to Cry3Bb1. Therefore, the most likely explanation is that these two factors, field-level selection and rootworm movement, both contributed to the observed uniformity of resistance to Cry3Bb1 corn.

Focal fields had grown more Cry3 corn and less non-Bt corn compared to surrounding fields (Table 4). Focal fields had experienced greater-than-expected injury to Cry3 corn in the past, so it is logical that a history of Cry3 corn cultivation would be reflected in the field management histories. Additionally, the proportion of Cry3 corn that had been grown in the past was positively correlated with both rootworm abundance and root injury (Table 5). Resistance to Cry3 is widespread in Iowa, and resistant populations may have high survival and impose high levels of injury to Cry3 corn [50,51]. This would suggest that abundance and injury would be higher in focal fields compared to surrounding fields, but we found that the two field types were equivalent. A factor that may partially explain the lack of a difference may be that surrounding fields had grown more non-Bt corn in the past (Table 4), creating a favorable habitat for rootworm, thus equalizing abundance and root injury between the field types. However, rootworm presence was low overall during this study, likely due to environmental factors including lower than average wintertime temperatures in 2014. Adult abundance in most fields was below the economic threshold calculated by Dunbar and Gassmann [52], and we found that injury to refuge (non-Bt) plants did not significantly differ from Bt plants. As a result, the influence of some aspects of field management on rootworm abundance and root injury in this study may have been obscured by low rootworm abundance. However, because focal fields grew more Cry3 corn and cultivation of this corn type was correlated with root injury and adult abundance, we would predict that root injury and abundance would have been higher in focal fields than in surrounding fields if rootworm presence had been higher. It is also notable that management in the year of sampling did not differ between these two field types (Fig 3), which may have contributed to similar levels of root injury and adult abundance. Additionally, management in other fields in the landscape not examined in this study may have influenced local populations. Thus, future work examining local landscapes may be beneficial for increasing our understanding of rootworm dynamics among fields.

The lack of a difference in root injury between Bt and non-Bt plants suggests that many farmers in this region did not derive an economic benefit from planting Bt corn with traits that target rootworm [21,22]. Rootworm populations were low overall during the years of this study, such that feeding injury by rootworm was below economic thresholds on Bt and non-Bt corn. If rootworm presence had been higher, the use of Bt corn could have reduced rootworm injury sufficiently to confer an economic benefit to farmers, as was observed in other fields in Iowa that were studied during the same period of time as the fields in this study [53]. Even though the use of Bt corn did not significantly reduce root injury, it was still exerting selection pressure on rootworm for Bt resistance. Such a situation is undesirable from the perspective of both integrated pest management and resistance management [54], and illustrates that planting Bt corn may be most economically beneficial in years when rootworm presence is sufficiently high. Farmers may be better served by monitoring adult populations, for example using commercially available adult monitoring traps, and planting Bt only in response to sufficiently large rootworm populations as described in Dunbar and Gassmann (52). Such an approach could increase profits for farmers while simultaneously lowering selection pressure for Bt resistance in the landscape.

As demonstrated here and elsewhere, resistance to Cry3Bb1 is now widespread throughout Iowa [55]. The first cases of resistance to Cry3Bb1 occurred in 2009 [24], and our study was conducted from 2015 to 2017, a maximum of six years between initial known resistance and practical ubiquity in eastern Iowa. Our results imply that gene flow of resistance may have occurred between fields with a history of injury to Cry3 corn and fields in the surrounding landscape during that period of time. However, the relative importance of the independent evolution of resistance compared to dispersal remains unclear. A model developed by Sisterson et al. [15] showed that Bt resistance can develop rapidly when resistance is non-recessive, irrespective of movement rate among fields. This may be of relevance to western corn rootworm, which exhibits non-recessive inheritance of resistance alleles [48]. Thus, further research in this area may be necessary to fully elucidate the relationship between field-evolved Bt resistance and movement in the landscape by western corn rootworm. Additionally, understanding the dynamic of resistance and movement will serve to clarify the relevant spatial scale for approaches of mitigating resistance to transgenic crops.

## Supporting information

S1 File (PDF)
