# Supplementary material for: Western corn rootworm abundance, injury to corn, and resistance to Cry3Bb1 in the local landscape of previous problem fields
Source: PLoS One. 2020 Jul 31;15(7):e0237094. doi: 10.1371/journal.pone.0237094 (PMC7394452; doi:10.1371/journal.pone.0237094)
Supplement: S1 File — (PDF) [file pone.0237094.s001.pdf]

Supplemental Material

Supplemental Table S1. Field cropping history for fields sampled in 2015.

Year	Field[1]	1	1a	1b	1c	1d	2[4]	3	4	4a	4b	4c
2015	Crop[2]	Corn	Corn	Corn	Corn	Corn	Corn	Corn	Corn	Corn	Corn	Corn
	Bt toxin[3]	5	4	4	4	4	0	4	1	0	5	4
	Soil	None	None	None	None	None	None	None	Yes	Yes	Yes	Yes
2014	Crop	Corn	Corn	Corn	Corn	Corn	Soy	Corn	Soy	Soy	Corn	Corn
	Bt toxin	5	4	4	4	4	.	4	.	.	4	3
	Soil	None	None	None	None	None	.	None	.	.	Yes	Yes
2013	Crop	Corn	Corn	Corn	Corn	Corn	Corn	Corn	Corn	Corn	Corn	Corn
	Bt toxin	5	0	0	4	4	Uk	4	5	5	0	5
	Soil	None	None	None	None	None	Uk	None	Yes	Yes	None	Yes
2012	Crop	Corn	Soy	Soy	Corn	Corn	Corn	Corn	Corn	Corn	Soy	Corn
	Bt toxin	5	.	.	4	4	Uk	1	3	3	.	4
	Soil	None	.	.	None	None	Uk	None	Yes	Yes	.	Yes
2011	Crop	Corn	Corn	Corn	Corn	Corn	Soy	Corn	Corn	Corn	Corn	Corn
	Bt toxin	0	4	4	4	4	.	Uk	Uk	Uk	4	4
	Soil	None	None	None	None	None	.	None	Yes	Yes	None	Yes
2010	Crop	Soy	Corn	Corn	Corn	Corn	Corn	Corn	Corn	Corn	Corn	Corn
	Bt toxin	.	4	4	4	4	Uk	Uk	Uk	Uk	Uk	4
	Soil	.	None	None	None	None	Uk	None	Yes	Yes	None	Yes

[1]Fields with numbers only are focal fields, and fields with a number and a letter are surrounding fields associated with the focal field number (e.g., Field 1 is a focal field, and Fields 1a, 1b, 1c, and 1d are its associated surrounding fields).
[2]Corn = Corn; Soy = Soybean; Alfa = Alfalfa; Uk = Unknown.
[3]Key for Bt toxins: 0 = no Bt targeting rootworm present; 1 = Cry3Bb1; 2 = Cry34/35Ab1; 3 = mCry3A; 4 = Cry3Bb1+Cry34/35Ab1; 5 = mCry3A+Cry34/35Ab1; Uk = Unknown.
[4]Excluded from multiple regression analysis.

Supplemental Table S1. (Continued) Field cropping history for fields sampled in 2015.

Year	Field[1]	4d[4]	5[4]	5a[4]	5b	5c[4]	5d
2015	**Crop[2]**	Corn	Corn	Corn	Corn	Corn	Corn
	**Bt toxin[3]**	4	0	0	4	4	4
	**Soil**	Yes	Yes	Yes	Yes	None	Yes
2014	**Crop**	Corn	Corn	Corn	Corn	Corn	Corn
	**Bt toxin**	Uk	Uk	Uk	4	Uk	4
	**Soil**	Uk	Uk	Uk	Yes	None	Yes
2013	**Crop**	Corn	Corn	Corn	Corn	Corn	Corn
	**Bt toxin**	Uk	Uk	Uk	4	Uk	4
	**Soil**	Uk	Uk	Uk	Yes	None	Yes
2012	**Crop**	Corn	Corn	Corn	Corn	Corn	Corn
	**Bt toxin**	Uk	Uk	Uk	4	Uk	4
	**Soil**	Uk	Uk	Uk	None	None	None
2011	**Crop**	Corn	Corn	Corn	Corn	Corn	Corn
	**Bt toxin**	Uk	Uk	Uk	4	Uk	4
	**Soil**	Uk	Uk	Uk	None	None	None
2010	**Crop**	Corn	Soy	Corn	Corn	Corn	Corn
	**Bt toxin**	Uk	.	Uk	4	Uk	4
	**Soil**	Uk	.	Uk	None	None	None

[1]Fields with numbers only are focal fields, and fields with a number and a letter are surrounding fields associated with the focal field number (e.g., Field 1 is a focal field, and Fields 1a, 1b, 1c, and 1d are its associated surrounding fields).
[2]Corn = Corn; Soy = Soybean; Alfa = Alfalfa; Uk = Unknown.
[3]Key for Bt toxins: 0 = no Bt targeting rootworm present; 1 = Cry3Bb1; 2 = Cry34/35Ab1; 3 = mCry3A; 4 = Cry3Bb1+Cry34/35Ab1; 5 = mCry3A+Cry34/35Ab1; Uk = Unknown.
[4]Excluded from multiple regression analysis.

Supplemental Table S2. Field cropping history for fields sampled in 2016.

Year	Field[1]	6	6a[4]	6b	6c	7	7a	7b	7c	8[4]	8a[4]	9
2016	Crop[2]	Corn	Corn	Corn	Corn	Corn	Corn	Corn	Corn	Corn	Corn	Corn
	Bt toxin[3]	4	2	4	5	4	2	0	5	1	3	1
	Soil	None	None	Yes	None	Yes	Yes	Yes	None	None	None	None
2015	Crop	Corn	Corn	Corn	Corn	Corn	Corn	Corn	Corn	Corn	Corn	Corn
	Bt toxin	4	Uk	4	2	4	4	4	5	Bt-Uk	Bt-Uk	0
	Soil	None	None	Yes	None	Yes	Yes	Yes	None	None	None	None
2014	Crop	Corn	Alfa	Corn	Corn	Corn	Corn	Corn	Soy	Corn	Corn	Corn
	Bt toxin	3	.	0	4	4	4	4	.	0	0	1
	Soil	None	.	None	None	Yes	Yes	Yes	.	None	None	None
2013	Crop	Corn	Alfa	Alfa	Corn	Soy	Corn	Corn	Corn	Soy	Soy	Corn
	Bt toxin	Uk	.	.	0	.	4	4	5	.	.	4
	Soil	None	.	.	None	.	Yes	Yes	None	.	.	Yes
2012	Crop	Corn	Alfa	Alfa	Corn	Corn	Corn	Corn	Corn	Corn	Corn	Corn
	Bt toxin	Uk	.	.	4	1	1	1	5	Bt-Uk	Bt-Uk	1
	Soil	None	.	.	None	None	None	None	None	None	None	None
2011	Crop	Corn	Alfa	Alfa	Corn	Corn	Soy	Soy	Corn	Corn	Corn	Corn
	Bt toxin	Uk	.	.	0	Uk	.	.	5	Bt-Uk	Bt-Uk	1
	Soil	Yes	.	.	None	None	.	.	None	None	None	None

[1]Fields with numbers only are focal fields, and fields with a number and a letter are surrounding fields associated with the focal field number (e.g., Field 1 is a focal field, and Fields 1a, 1b, 1c, and 1d are its associated surrounding fields).
[2]Corn = Corn; Soy = Soybean; Alfa = Alfalfa; Uk = Unknown.
[3]Key for Bt toxins: 0 = no Bt targeting rootworm present; 1 = Cry3Bb1; 2 = Cry34/35Ab1; 3 = mCry3A; 4 = Cry3Bb1+Cry34/35Ab1; 5 = mCry3A+Cry34/35Ab1; Uk = Unknown.
[4]Excluded from multiple regression analysis.

Supplemental Table S2. (Continued) Field cropping history for fields sampled in 2016.

Year	Field[1]	9a	9b	9c	10	10a	11	11a	11b[4]	11c[4]	11d[4]
2016	**Crop[2]**	Corn	Corn	Corn	Corn	Corn	Corn	Corn	Corn	Corn	Corn
	**Bt toxin[3]**	0	3	4	1	4	4	4	1	4	4
	**Soil**	None	Yes	None	None	None	None	None	None	None	None
2015	**Crop**	Soy	Corn	Corn	Corn	Soy	Corn	Corn	Soy	Corn	Soy
	**Bt toxin**	.	3	4	4	.	4	4	.	Uk	.
	**Soil**	.	Yes	None	Yes	.	None	None	.	Uk	.
2014	**Crop**	Corn	Corn	Soy	Corn	Corn	Corn	Corn	Corn	Corn	Corn
	**Bt toxin**	3	3	.	4	4	4	4	Uk	Uk	Uk
	**Soil**	None	None	.	None	None	None	None	Uk	Uk	Uk
2013	**Crop**	Soy	Corn	Corn	Corn	Corn	Corn	Corn	Corn	Corn	Corn
	**Bt toxin**	.	3	Uk	4	4	4	Uk	Uk	Uk	Uk
	**Soil**	.	None	Uk	None	None	None	None	Uk	Uk	Uk
2012	**Crop**	Corn	Corn	Soy	Corn	Corn	Corn	Corn	Soy	Corn	Corn
	**Bt toxin**	3	3	.	4	4	4	Uk	.	Uk	Uk
	**Soil**	None	None	.	None	None	None	None	.	Uk	Uk
2011	**Crop**	Soy	Soy	Corn	Corn	Soy	Corn	Corn	Corn	Corn	Soy
	**Bt toxin**	.	.	Uk	1	.	4	Uk	Uk	Uk	.
	**Soil**	.	.	Uk	None	.	None	None	Uk	Uk	.

[1]Fields with numbers only are focal fields, and fields with a number and a letter are surrounding fields associated with the focal field number (e.g., Field 1 is a focal field, and Fields 1a, 1b, 1c, and 1d are its associated surrounding fields).
[2]Corn = Corn; Soy = Soybean; Alfa = Alfalfa; Uk = Unknown.
[3]Key for Bt toxins: 0 = no Bt targeting rootworm present; 1 = Cry3Bb1; 2 = Cry34/35Ab1; 3 = mCry3A; 4 = Cry3Bb1+Cry34/35Ab1; 5 = mCry3A+Cry34/35Ab1; Uk = Unknown.
[4]Excluded from multiple regression analysis.

Supplemental Table S3. Field cropping history for fields sampled in 2017.

Year	Field[1]	12	12a	12b	13	13a[4]	13b	13c	14[4]	14a[4]	14b	14c
**2017**	**Crop[2]**	Corn	Corn	Corn	Corn	Corn	Corn	Corn	Corn	Corn	Corn	Corn
	**Bt toxin[3]**	4	0	0	5	0	0	4	1	1	0	4
	**Soil**	Yes	None	Yes	Yes	None	None	None	None	None	None	None
**2016**	**Crop**	Corn	Soy	Soy	Corn	Soy	Soy	Corn	Soy	Soy	Soy	Corn
	**Bt toxin**	0	.	.	3	.	.	0	.	.	.	4
	**Soil**	Yes	.	.	Yes	.	.	None	.	.	.	None
**2015**	**Crop**	Soy	Corn	Corn	Corn	Corn	Corn	Soy	Corn	Corn	Corn	Corn
	**Bt toxin**	.	0	0	3	3	4	.	Uk	Uk	0	4
	**Soil**	.	None	Yes	Yes	Uk	None	.	None	None	None	None
**2014**	**Crop**	Corn	Soy	Corn	Corn	Soy	Corn	Corn	Soy	Soy	Soy	Corn
	**Bt toxin**	4	.	0	3	.	0	4	.	.	.	4
	**Soil**	None	.	Yes	Yes	.	None	None	.	.	.	None
**2013**	**Crop**	Corn	Corn	Soy	Corn	Corn	Soy	Corn	Corn	Corn	Corn	Soy
	**Bt toxin**	1	0	.	3	Uk	.	0	Uk	Uk	Uk	.
	**Soil**	None	None	.	Yes	Uk	.	None	None	None	None	.
**2012**	**Crop**	Corn	Soy	Corn	Corn	Soy	Corn	Soy	Soy	Soy	Corn	Corn
	**Bt toxin**	1	.	0	0	.	4	.	.	.	Uk	4
	**Soil**	None	.	None	None	.	None	.	.	.	None	None

[1]Fields with numbers only are focal fields, and fields with a number and a letter are surrounding fields associated with the focal field number (e.g., Field 1 is a focal field, and Fields 1a, 1b, 1c, and 1d are its associated surrounding fields).

[2]Corn = Corn; Soy = Soybean; Alfa = Alfalfa; Uk = Unknown.

[3]Key for Bt toxins: 0 = no Bt targeting rootworm present; 1 = Cry3Bb1; 2 = Cry34/35Ab1; 3 = mCry3A; 4 = Cry3Bb1+Cry34/35Ab1; 5 = mCry3A+Cry34/35Ab1; Uk = Unknown.

[4]Excluded from multiple regression analysis.

Supplemental Table S3. (Continued) Field cropping history for fields sampled in 2017.

Year	Field[1]	15	15a	15b	15c	16	16a	17	17a	18[4]	18a[4]	18b[4]
2017	**Crop[2]**	Corn	Corn	Corn	Corn	Corn	Corn	Corn	Corn	Corn	Corn	Corn
	**Bt toxin[3]**	4	4	4	4	4	4	4	4	4	5	2
	**Soil**	None	None	None	Yes	None	None	None	None	None	None	None
2016	**Crop**	Corn	Corn	Soy	Corn	Corn	Corn	Corn	Corn	Corn	Corn	Corn
	**Bt toxin**	4	0	.	4	4	0	4	4	Uk	Uk	Uk
	**Soil**	None	None	.	Yes	Yes	None	None	None	None	Uk	Uk
2015	**Crop**	Soy	Soy	Corn	Corn	Corn	Soy	Corn	Corn	Corn	Corn	Soy
	**Bt toxin**	.	.	4	4	0	.	4	4	Uk	Uk	.
	**Soil**	.	.	None	Yes	None	.	None	None	None	Uk	.
2014	**Crop**	Corn	Corn	Soy	Corn	Soy	Corn	Soy	Soy	Soy	Soy	Corn
	**Bt toxin**	4	0	.	4	.	2	.	.	.	.	Uk
	**Soil**	Yes	None	.	Yes	.	None	.	.	.	.	Uk
2013	**Crop**	Corn	Soy	Corn	Corn	Corn	Corn	Soy	Soy	Corn	Corn	Corn
	**Bt toxin**	4	.	4	4	2	Uk	.	.	Uk	Uk	Uk
	**Soil**	Yes	.	None	Yes	Yes	None	.	.	Yes	Uk	Uk
2012	**Crop**	Soy	Corn	Soy	Corn	Corn	Corn	Corn	Corn	Corn	Corn	Corn
	**Bt toxin**	.	0	.	4	1	0	4	4	Uk	Uk	Uk
	**Soil**	.	None	.	Yes	None	None	None	None	Yes	Uk	Uk

[1]Fields with numbers only are focal fields, and fields with a number and a letter are surrounding fields associated with the focal field number (e.g., Field 1 is a focal field, and Fields 1a, 1b, 1c, and 1d are its associated surrounding fields).

[2]Corn = Corn; Soy = Soybean; Alfa = Alfalfa; Uk = Unknown.

[3]Key for Bt toxins: 0 = no Bt targeting rootworm present; 1 = Cry3Bb1; 2 = Cry34/35Ab1; 3 = mCry3A; 4 = Cry3Bb1+Cry34/35Ab1; 5 = mCry3A+Cry34/35Ab1; Uk = Unknown.

[4]Excluded from multiple regression analysis.

Supplemental Table S3. (Continued) Field cropping history for fields sampled in 2017.

Year	Field	18c[4]	19[4]	19a[4]	20	20a	20b
2017	**Crop**	Corn	Corn	Corn	Corn	Corn	Corn
	**Bt toxin**	0	4	0	0	0	4
	**Soil**	None	None	None	None	None	Yes
2016	**Crop**	Corn	Corn	Soy	Soy	Soy	Corn
	**Bt toxin**	Uk	Uk	.	.	.	4
	**Soil**	Uk	None	.	.	.	None
2015	**Crop**	Soy	Soy	Corn	Corn	Corn	Corn
	**Bt toxin**	.	.	Uk	4	4	4
	**Soil**	.	.	None	None	None	None
2014	**Crop**	Corn	Corn	Soy	Corn	Corn	Corn
	**Bt toxin**	Uk	Uk	.	4	4	4
	**Soil**	Uk	None	.	None	None	None
2013	**Crop**	Corn	Corn	Corn	Soy	Soy	Corn
	**Bt toxin**	Uk	Uk	Uk	.	.	1
	**Soil**	Uk	None	None	.	.	None
2012	**Crop**	Corn	Corn	Soy	Corn	Corn	Corn
	**Bt toxin**	Uk	Uk	.	1	1	Uk
	**Soil**	Uk	None	.	None	None	None

[1]Fields with numbers only are focal fields, and fields with a number and a letter are surrounding fields associated with the focal field number (e.g., Field 1 is a focal field, and Fields 1a, 1b, 1c, and 1d are its associated surrounding fields).
[2]Corn = Corn; Soy = Soybean; Alfa = Alfalfa; Uk = Unknown.
[3]Key for Bt toxins: 0 = no Bt targeting rootworm present; 1 = Cry3Bb1; 2 = Cry34/35Ab1; 3 = mCry3A; 4 = Cry3Bb1+Cry34/35Ab1; 5 = mCry3A+Cry34/35Ab1; Uk = Unknown.
[4]Excluded from multiple regression analysis.

Supplemental Table S4. Correlation matrix of potential variables for multiple regression analysis.

**Variable**	1	2	3	4	5	6	7	8	9	10	11	12	13	14
1	1	-	-	-	-	-	-	-	-	-	-	-	-	-
2	-0.22	1	-	-	-	-	-	-	-	-	-	-	-	-
3	-0.12	-0.09	1	-	-	-	-	-	-	-	-	-	-	-
4	-0.65	-0.48	-0.26	1	-	-	-	-	-	-	-	-	-	-
5	0.05	-0.06	0.02	-0.02	1	-	-	-	-	-	-	-	-	-
6	-0.29	-0.07	-0.10	0.34	0.26	1	-	-	-	-	-	-	-	-
7	-0.33	-0.04	-0.16	0.38	0.30	0.75	1	-	-	-	-	-	-	-
8	0.04	-0.02	0.10	-0.06	**0.86**	0.10	0.25	1	-	-	-	-	-	-
9	0.63	-0.14	-0.11	-0.40	-0.11	-0.47	-0.51	-0.10	1	-	-	-	-	-
10	0.13	0.64	0.02	-0.50	0.28	-0.07	0.08	0.32	-0.13	1	-	-	-	-
11	-0.16	-0.09	0.47	0.05	-0.06	-0.05	0.11	0.02	0.08	-0.08	1	-	-	-
12	-0.56	-0.29	-0.01	0.67	-0.11	0.43	0.32	-0.17	-0.71	-0.58	-0.17	1	-	-
13	-0.13	0.09	0.12	0.00	**0.80**	0.17	0.31	**0.94**	-0.26	0.33	0.06	-0.06	1	-
14	0.42	-0.10	-0.05	-0.24	0.37	-0.19	-0.06	0.38	0.41	0.00	-0.08	-0.32	0.03	1

[a] Variable numbers are:

1 Non -Bt corn (year of sampling)
2 Cry3 corn (year of sampling)
3 Cry34/35Ab1 corn (year of sampling)
4 Pyramided corn (year of sampling)
5 Soil insecticide use (year of sampling)
6 Years of continuous corn
7 Proportion of years corn grown in 6 years
8 Proportion of years soil insecticide was used in 6 years
9 Proportion of years non-Bt corn was grown in 6 years
10 Proportion of years Cry3 corn was grown in 6 years
11 Proportion of years Cry34/35Ab1 was grown in 6 years
12 Proportion of years pyramided corn was grown in 6 years
13 Proportion of years soil insecticide was used on Bt corn in 6 years
14 Proportion of years soil insecticide was used on non-Bt corn in 6 years

[b] Variables 5 and 13 were excluded from multiple regression analysis due to collinearity with other variable
